# A Natural Chimeric *Pseudomonas* Bacteriocin with Novel Pore-Forming Activity Parasitizes the Ferrichrome Transporter

**DOI:** 10.1128/mBio.01961-16

**Published:** 2017-02-21

**Authors:** Maarten G. K. Ghequire, Lieselore Kemland, Ernesto Anoz-Carbonell, Susan K. Buchanan, René De Mot

**Affiliations:** a Centre of Microbial and Plant Genetics, KU Leuven, Heverlee, Belgium; b Laboratory of Molecular Biology, National Institute of Diabetes and Digestive and Kidney Diseases, National Institutes of Health, Bethesda, Maryland, USA; University of Nebraska

## Abstract

Modular bacteriocins represent a major group of secreted protein toxins with a narrow spectrum of activity, involved in interference competition between Gram-negative bacteria. These antibacterial proteins include a domain for binding to the target cell and a toxin module at the carboxy terminus. Self-inhibition of producers is provided by coexpression of linked immunity genes that transiently inhibit the toxin’s activity through formation of bacteriocin-immunity complexes or by insertion in the inner membrane, depending on the type of toxin module. We demonstrate strain-specific inhibitory activity for PmnH, a *Pseudomonas* bacteriocin with an unprecedented dual-toxin architecture, hosting both a colicin M domain, potentially interfering with peptidoglycan synthesis, and a novel colicin N-type domain, a pore-forming module distinct from the colicin Ia-type domain in *Pseudomonas aeruginosa* pyocin S5. A downstream-linked gene product confers PmnH immunity upon susceptible strains. This protein, ImnH, has a transmembrane topology similar to that of *Pseudomonas* colicin M-like and pore-forming immunity proteins, although homology with either of these is essentially absent. The enhanced killing activity of PmnH under iron-limited growth conditions reflects parasitism of the ferrichrome-type transporter for entry into target cells, a strategy shown here to be used as well by monodomain colicin M-like bacteriocins from pseudomonads. The integration of a second type of toxin module in a bacteriocin gene could offer a competitive advantage against bacteria displaying immunity against only one of both toxic activities.

## IMPORTANCE

In their continuous struggle for ecological space, bacteria face a huge load of contenders, including phylogenetically related strains that compete for the same niche. One important group of secreted antibacterial proteins assisting in eliminating these rivals are modular bacteriocins of Gram-negative bacteria, comprising a domain for docking onto the cell envelope of a target cell, a translocation domain enabling subsequent cellular entry, and a toxin module that kills target cells via enzymatic or pore-forming activity. We here demonstrate the antagonistic function of a *Pseudomonas* bacteriocin with unique architecture that combines a putative enzymatic colicin M-like domain and a novel pore-forming toxin module. For target cell recognition and entry, this bacteriocin hybrid takes advantage of the ferrichrome transporter, also parasitized by enzymatic *Pseudomonas* bacteriocins devoid of the pore-forming module. Bacteriocins with an expanded toxin potential may represent an inventive bacterial strategy to alleviate immunity in target cells.

## INTRODUCTION

The metabolically versatile genus *Pseudomonas* is able to colonize very diverse competitive niches and includes a tremendous variety of organisms ranging from opportunistic pathogens to plant growth-promoting bacteria and environmental pollutant degraders (1, 2). To face competitors, pseudomonads are armed with an arsenal of antagonistic molecules, diverse both from a structural point of view and in mechanism of action. A subset of these *Pseudomonas* compounds are bacteriocins, secreted ribosomally synthesized antibacterial peptides and proteins that selectively kill related bacteria (3). In *Pseudomonas aeruginosa* in particular, bacteriocins—there called pyocins—have been associated with an ecoevolutionary advantage for producer strains (4–7).

Several classes of *Pseudomonas* bacteriocins have been identified. Together with the multisubunit cell-perforating tailocins (8), S-type bacteriocins constitute a very abundant group in *Pseudomonas* genomes (3). These modular polypeptides resemble the colicins from *Escherichia coli* (9) and share a general organization that includes a domain for target cell attachment, a domain mediating subsequent translocation, and a carboxy-terminal toxin domain. The latter may display nuclease activity, as, for example, found in pyocins S1 (DNase), S4 (tRNase), and S6 (rRNase), or possess pore-forming function, as in pyocin S5 (PyoS5) (3, 10). Less widespread in pseudomonads are M-type bacteriocins (PseuMs), equipped with a colicin M (ColM) domain and exerting enzymatic activity in the periplasm, via cleavage of the lipid II peptidoglycan precursor (11–13).

Self-inhibition of strains producing modular pyocins is prevented by coexpression of an immunity gene, located downstream of the bacteriocin gene on the same or opposite strand. These immunity proteins shield the toxin part of the bacteriocins by formation of toxin-immunity complexes, as structurally elucidated for the nucleases of pyocins S2 and AP41 (14). Lethality due to pore formation by pyocin S5 (PyoS5) is transiently impeded along the secretory route via a membrane-integrated protein consisting of three transmembrane helices (TMHs) (15). PseuM immunity is also delivered by a membrane-integrated protein, called PmiA, consisting of four TMHs but showing no sequence similarity to the PyoS5 immunity protein (16). In contrast to other immunity proteins protecting from enzymatic toxin domains, PmiA proteins display very poor sequence homology with the exception of a short semiconserved periplasmic motif, whose role in the PseuM immunity mechanism remains elusive at this point.

The target for bacteriocin docking onto cells has been identified for several S pyocins. It typically concerns outer membrane proteins (OMPs) involved in the uptake of iron via siderophores: type I ferripyoverdine transporter FpvAI for pyocins S2, S4, and SD2 (17–19), type II ferripyoverdine transporter FpvAII for pyocin S3 (20), and ferripyochelin receptor FptA for pyocin S5 (21). Some S pyocins carry (almost) identical amino-terminal regions and basically differ only in their toxin-immunity module, emphasizing the peculiar modularity of these allelopathic molecules. Although omnipresent in *Pseudomonas* genomes (3), few S-type bacteriocins have been functionally characterized in other *Pseudomonas* species (22). To date, their partner mediating initial binding and also the one(s) of PseuMs remain unknown. Since significant structural similarity was observed between the PseuMs from *P. aeruginosa* and *Pseudomonas syringae* and colicin M from *E. coli*, it was suggested that these bacteriocins share a common ancestry and may target the same type of cell surface component (12, 13, 23).

In this study, we demonstrate the antagonistic functionality of a unique *Pseudomonas* bacteriocin that hosts two toxin modules. This bacteriocin carries a ColM domain and is followed by a novel type of pore-forming domain, not previously described in *Pseudomonas*. The role of a gene located downstream of the bacteriocin in providing immunity is also explored. We further show that this bacteriocin targets a TonB-dependent OMP that shares homology with the ferrichrome transporter from *P. aeruginosa*. We also demonstrate that two different PseuM bacteriocins equally target this surface-exposed protein.

## RESULTS

### PmnH combines an enzymatic domain and a novel type of pore-forming module.

**FIG 1:**
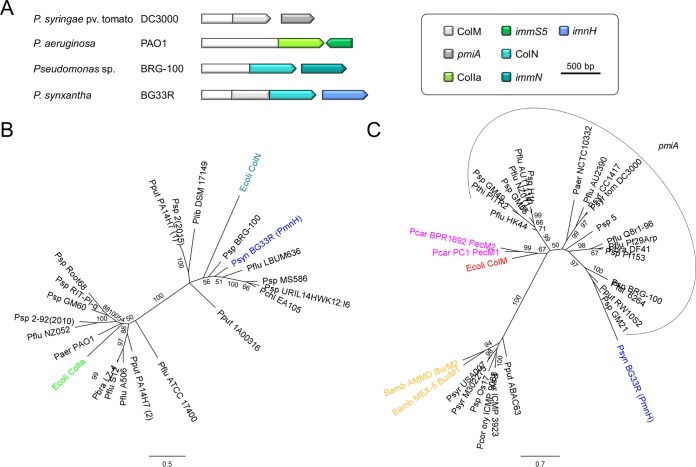
(A) Schematic gene organizations of representative ColM- and pore domain-containing bacteriocin genes and (putative) immunity genes in pseudomonads. Arrows correspond with gene orientations. The color key outlines the different bacteriocin domains and immunity gene type. (B and C) Maximum likelihood phylogenetic trees of pore-forming domains (B) and ColM domains (C) from (hypothetical) *Pseudomonas* bacteriocins and characterized betaproteobacterial/gammaproteobacterial bacteriocins. Highly homologous domains (>95% pairwise amino acid identity) are not included. The scale bars represent 0.5 (B) and 0.7 (C) substitutions per site. Bootstrap values (percentages of 1,000 replicates) higher than 50 are shown at the branches. PmnH from *Pseudomonas synxantha* BG33R was selected as a PmnH representative, and its corresponding pore-forming and ColM domains are colored blue; colicin Ia (ColIa), colicin N (ColN), and colicin M (ColM) domains from *E. coli* are shown in green, teal, and red, respectively; ColM domains from *Pectobacterium* and *Burkholderia* are shown in pink and orange, respectively. (B) *P. putida* PA14H7 encodes two different pore-forming bacteriocins, one equipped with a ColN-type domain (1) and one with a ColIa-type domain (2). (C) *Pseudomonas* PseuM bacteriocins followed by a (predicted) *pmiA*-type immunity gene are grouped by an arc. Abbreviations for species names: Bamb, *Burkholderia ambifaria*; Ecoli, *Escherichia coli*; Paer, *Pseudomonas aeruginosa*; Pbra, *Pseudomonas brassicacearum*; Pcar, *Pectobacterium carotovorum*; Pchl, *Pseudomonas chlororaphis*; Pcor ory, *Pseudomonas coronafaciens* pv. oryzae; Pflu, *Pseudomonas fluorescens*; Plib, *Pseudomonas libanensis*; Pput, *Pseudomonas putida*; Psp, *Pseudomonas* sp.; Psyn, *Pseudomonas synxantha*; Psyr (tom), *Pseudomonas syringae* (pv. tomato); Pthi, *Pseudomonas thivervalensis*; Ptol, *Pseudomonas tolaasii*.

Known modular bacteriocins host a single toxin domain and are accompanied by a cognate immunity protein to prevent self-inhibition. However, genome analysis in pseudomonads has revealed genes encoding putative bacteriocins with two nuclease modules at their C termini: the “hybrids” PsdH1 and PsdH2 are typified by two S1-like DNase domains and by an S1-like and S3-like DNase domain, respectively (3). Yet another hybrid organization, comprising two apparently unrelated modules, is found in PmnH, joining a ColM domain (Pfam PF14859) to a pore-forming domain (Pfam PF01024) (Fig. 1A) (24). This pore-forming domain is phylogenetically distinct from the domain in pyocin S5 (PyoS5) (Fig. 1B; see also Fig. S1 in the supplemental material): the latter domain is more closely related to colicin Ia (ColIa) (25), whereas PmnH’s C-terminal domain belongs to a clade with colicin N (ColN) (26, 27), sharing 44.6% and 33.5% amino acid identity to the corresponding colicin domains, respectively. The ColIa-type pore-forming domain of pyocin S5 is present in ~28% of the sequenced *P. aeruginosa* genomes (draft and full genomes), whereas the ColN-type pore-forming domain can be retrieved only in other *Pseudomonas* species. Overall, (putative) pore-forming bacteriocins, of either the ColIa or the ColN type, are very rare in *Pseudomonas* species other than *P. aeruginosa*, and they are encoded in only a few strains (24 isolates versus >800 draft and full genomes). The opposite was previously observed for rRNase bacteriocins that are rare in *P. aeruginosa* but very abundant in other *Pseudomonas* species (3).

10.1128/mBio.01961-16.1 FIG S1  Multiple sequence alignment of pore-forming domains in (putative) *Pseudomonas* bacteriocins and pore-forming domains of colicins Ia and N. Abbreviations for species names are the same as in Fig. 1. Gray shading reflects the degree of conservation. Highly homologous pore domains (>95% pairwise amino acid identity) are not included in the alignment. The PmnH from *P. synxantha* BG33R was taken as a PmnH representative. Download FIG S1, TIF file, 2.6 MB. Copyright © 2017 Ghequire et al. 2017 Ghequire et al. This content is distributed under the terms of the Creative Commons Attribution 4.0 International license.

The ColM domain of PmnH contains several of the coordinating residues previously associated with structural integrity and catalytic activity in colicin M from *E. coli* and PseuMs from *P. aeruginosa* and *P. syringae* (Fig. S2) (12, 13, 28). The equivalent of the *E. coli* pivotal motif DxYD(x_5_)HR in the carboxy-terminal part of the ColM module (residues 226 to 236) is present in nearly all PmnH proteins as HxYD(x_5_)FK (positions 224 to 234, with x representing any amino acid). The His224 and Lys234 residues, deviating from the prototypical ColM motif, are not present in any other pseudomonad ColM domain, in contrast to the frequently present Phe233 (such as in PseuMs from *Pseudomonas* sp. strain GM21 and *Pseudomonas tolaasii* 6264). Among the PmnH-encoding strains, the equivalent sequence in *Pseudomonas* sp. strain 25R14 [IxYT(x_5_)LK] (29) deviates substantially from the ColM-derived consensus motif. A diverged ColM motif not affecting bactericidal activity was previously observed for M-type burkhocins, for example as DxYK(x_5_)-R in BurM1 of *Burkholderia ambifaria* MEX-5 (30). In this case, possible lipid II hydrolase activity was not examined. The amino acid sequence of the ColM domain of PmnH clusters with those of the equivalent domains of PseuMs, being protected by PmiA immunity proteins (Fig. 1C) (16).

10.1128/mBio.01961-16.2 FIG S2  Multiple sequence alignment of ColM domains in (putative) *Pseudomonas* bacteriocins and characterized bacteriocins from other gammaproteobacteria. Abbreviations for species names are the same as in Fig. 1. Gray shading reflects the degree of conservation. Highly homologous ColM domains (>95% pairwise amino acid identity) are not included in the alignment. The ColM motif, presumed to be involved in the lipid II hydrolase activity, is boxed in blue. The PmnH from *P. synxantha* BG33R was taken as a PmnH representative. Download FIG S2, TIF file, 2.4 MB. Copyright © 2017 Ghequire et al. 2017 Ghequire et al. This content is distributed under the terms of the Creative Commons Attribution 4.0 International license.

**FIG 2:**
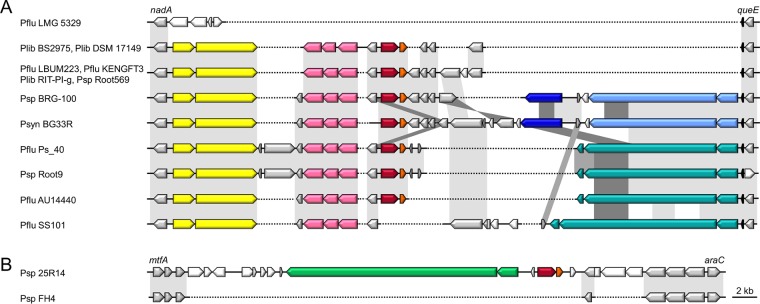
Genetic organization of the *pmnH* loci in *Pseudomonas* spp. Synteny is visualized by sequence conservation (gray shading, darker gray in cases of overlap) for the genes (shown as arrows) located between the orthologs *nadA* (quinolinate synthase) and *queE* (7-carboxy-7-deazaguanine synthase; not syntenic in *Pseudomonas* sp. Root9) (A) and between *mtfA* (zinc-dependent peptidase) and *araC* (AraC family transcriptional regulator) (B). The *pmnH* gene and *imnH* gene are colored dark red and light red, respectively. Syntenic genes/gene fragments are gray; nonsyntenic genes/gene fragments are white. Genes encoding a putative tripartite efflux pump are pink, and a tRNA-Lys gene is black. Genes encoding a TPS system for a hemagglutinin repeat protein located upstream of *pmnH-imnH* are shown in yellow (A) and green (B), those encoding the protein downstream of *pmnH-imnH* are in light blue, and fragmented hemagglutinin repeat genes are in dark blue. Gene triplets constituting a putative pseudomonad class V CDI system (31) are in teal. Dotted lines indicate the lack of equivalent nucleotide sequence. In *Pseudomonas* sp. 25R14, *pmnH* and *imnH* have been recruited to a different locus. Loci from *P. fluorescens* strains LMG 5329 and SS101 (*nadA-queE*) and *Pseudomonas* sp. strain FH4 (*mtfA-araC*) were added for comparison to similar regions lacking the *pmnH-imnH* gene couple. *Pseudomonas* species abbreviations are the same as in Fig. 1.

The *pmnH* gene (1,389 bp) occurs in isolates from several *Pseudomonas* species, originating from diverse sources such as rhizosphere, water, and cystic fibrosis lung: *Pseudomonas fluorescens* (AU14440, LBUM223, KENGFT3, and Ps_40), *Pseudomonas libanensis* (BS2975, DSM 17149, and RIT-PI-g), *Pseudomonas synxantha* (BG33R), and other *Pseudomonas* sp. strains (25R14, BRG-100, Root9, and Root569). The encoded PmnH sequences show near-perfect conservation (~98% pairwise amino acid identity), with the notable exception of the more diverged 25R14 protein (~59% amino acid identity). The highly conserved *pmnH* genes are part of a stretch of ~2.5 kb with lower-than-average GC content (~48% versus ~61%) and reside in a semivariable context with strain-dependent differences, suggesting that the *pmnH*-flanking sequences have been subject to various rearrangements (Fig. 2). The region upstream of *pmnH* encodes a putative tripartite efflux pump of the major facilitator family (opposite strand) and a putative two-partner secretion (TPS) system for a hemagglutinin repeat protein (same strand). In a number of strains, a second type of TPS system is present further downstream of *pmnH* (opposite strand), sometimes joined by a shorter, partially homologous hemagglutinin repeat gene (possibly a fragmented gene remnant). The presence of Pfam domain DUF637 with a conserved NEALL motif in the proteins encoded by the downstream hemagglutinin repeat genes (detected in strains AU14440, Root9, and Ps_40) suggests that these TPS systems may be part of a contact-dependent inhibition (CDI) cassette (31). In addition to bacteriocins, CDI systems play key roles in interbacterial competition, although confined to inhibition of neighboring cells (32). In closer proximity to *pmnH*, a number of genes without known function arise, as well as more unique interspersed genes (Fig. 2). The more divergent *pmnH* homologue from *Pseudomonas* sp. 25R14 has been recruited to a different but also poorly conserved region, equally harboring a TPS system but distinct from those found in the other *pmnH-*carrying strains. Notably, some of the *pmnH*-carrying strains also encode a “regular” single-toxin form of bacteriocin with the same, ColN-type pore-forming domain (*Pseudomonas* sp. strain BRG-100) or of the ColIa/PyoS5 type (*P. libanensis* RIT-PI-g). Both types of monofunctional bacteriocin genes are found in the *pmnH*-lacking *Pseudomonas putida* PA14H7, as cargo of its tailocin (8).

### PmnH antagonistic activity requires the pore-forming domain.

**FIG 3:**
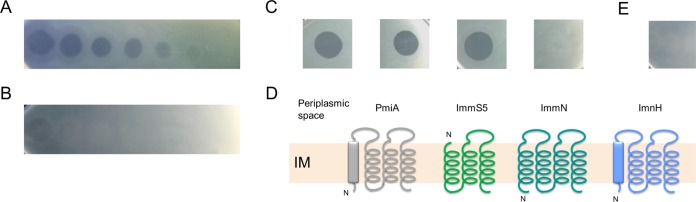
(A) Spot assay of 5-fold serial dilutions of PmnH against susceptible strain *P. fluorescens* LMG 1794 on CAA. (B) Spot assay of 5-fold serial dilutions of PmnH against susceptible strain *P. fluorescens* LMG 1794 on CAA supplemented with FeCl_3_ (50 µM). (C) Spot assay against *P. fluorescens* LMG 1794 with—from left to right—PmnH, PmnH(H224A), PmnH(D227A), and PmnHΔColN. (D) Schematic representation of membrane-integrated bacteriocin immunity genes in *Pseudomonas*. Color code is similar to that in Fig. 1A. Sec-dependent signal peptides, if present in the majority of the cases, are shown in a box. IM, inner membrane. (E) Spot assay of PmnH against susceptible strain LMG 1794 equipped with *imnH*.

Among the highly conserved* pmnH* genes, the one from *P. synxantha* BG33R (encoding PSEBG33_RS05990) was selected for further characterization. Previously, it was found that a short proline-rich sequence at the amino terminus of the PseuM from a *P. syringae* pathovar is required for cytotoxicity (13), and a similar semiconserved sequence was detected in ColM-like bacteriocins from other *Pseudomonas* species (16) and *Burkholderia* (30). Such a sequence may act as a functional equivalent of the TonB box from colicin M in *E. coli* and is equally present in the gene product of *pmnH* (Fig. S3). To avoid interference of the His tag with the possible import-related function of this sequence, *pmnH* was cloned to encode a C-terminal tag. After recombinant expression and purification, antagonistic activity of His-tagged PmnH was evaluated against a panel of *Pseudomonas* strains (type strains and a selection of in-house environmental isolates, *n* = 35), of which ~11% proved susceptible (Table S1). Susceptibility of the indicator strains markedly increased when bacteria were grown in and plated on iron-poor media such as King’s B and Casamino Acids medium (CAA) (Fig. 3A). On the other hand, when the strains were grown on CAA in the presence of FeCl_3_ (50 µM), growth inhibition was almost completely annihilated (Fig. 3B), suggesting that PmnH targets a protein that is upregulated under iron-poor conditions.

10.1128/mBio.01961-16.3 FIG S3  Multiple sequence alignment of amino-terminal sequences preceding the ColM domain in colicin M, characterized PseuMs, and PmnH. Gray shading reflects the degree of conservation. Species abbreviations are as in Fig. 1. Putative TonB-binding boxes are indicated in orange. The equivalent motif in the sequence-diverged PmnH from *Pseudomonas* sp. 25R14 is present as MSTELPALV. Download FIG S3, TIF file, 0.3 MB. Copyright © 2017 Ghequire et al. 2017 Ghequire et al. This content is distributed under the terms of the Creative Commons Attribution 4.0 International license.

10.1128/mBio.01961-16.6 TABLE S1  Antibacterial activity of purified recombinant PmnH against *Pseudomonas* spp.: +, susceptible (clear halo); T, turbid (semitransparent halo); −, no halo. Download TABLE S1, DOCX file, 0.01 MB. Copyright © 2017 Ghequire et al. 2017 Ghequire et al. This content is distributed under the terms of the Creative Commons Attribution 4.0 International license.

To evaluate whether one or both toxin modules contribute to growth inhibition of PmnH-susceptible strains, truncated forms without the ColN domain and forms with an H224A or D227A mutation in the ColM domain were constructed and produced. The former construct retains only the ColM domain, whereas the point mutants keep only the functionality of the pore-forming domain, based on previous studies on abrogated lipid II hydrolase activity of ColM domain mutants (12, 13, 28, 33). All indicator strains remained susceptible to the point mutant variants [PmnH(H224A) and PmnH(D227A)] (Fig. 3C), indicative of killing due to pore formation. No strain susceptible to the pore-forming domain deletion (PmnHΔColN) could yet be identified.

### PmnH immunity is conferred by a downstream-coupled gene.

Based on previous observations for modular pyocins and colicins, it was anticipated that (a) dedicated immunity partner(s) would likely be encoded in close proximity to *pmnH*. No homologue of *cmi*, the prototypical ColM immunity gene of *E. coli* (34), was found, but a different gene encoding a protein product of 182 to 184 amino acids (aa) (~84% pairwise identity among homologues), further designated ImnH, was conserved immediately downstream of *pmnH* (on the same strand) (Fig. 1A). In line with its more divergent toxin, the corresponding linked protein of *Pseudomonas* sp. 25R14 (182 aa) also shows a lower degree of amino acid sequence identity (~60%). The ImnH proteins lack a known protein domain and host four predicted TMHs, the first of which may act as a Sec-dependent signal sequence. PseuM immunity proteins (PmiAs) equally carry four TMHs, the first of which is equally predicted to act as a signal sequence, but these proteins are considerably smaller (~137 aa) (16) and cannot meaningfully be aligned with ImnHs. Notably, putative immunity partners of “mono” ColN-like pore formers (ImmNs) in pseudomonads also bear four TMHs and have about the same size as ImnHs (~187 aa) (Fig. 3D). These membrane proteins share borderline homology with ImnHs (Fig. S4) and are encoded on the same strand as the bacteriocin genes, unlike the gene organization of ColIa-type pore-forming bacteriocins (Fig. 1A). *E. coli*’s colicin N immunity protein (Cni) (35, 36), although slightly smaller (174 aa), is equally constituted of 4 TMHs and shares ~20% amino acid identity with ImnH proteins.

10.1128/mBio.01961-16.4 FIG S4  Multiple sequence alignment of putative ColN-type immunity proteins and ImnH. Differential gray shading indicates the degree of conservation. Species abbreviations are as in Fig. 1. Predicted transmembrane regions are boxed in blue, and Sec-dependent signal peptides, if present, are in red. Download FIG S4, TIF file, 1.1 MB. Copyright © 2017 Ghequire et al. 2017 Ghequire et al. This content is distributed under the terms of the Creative Commons Attribution 4.0 International license.

The *imnH* gene of *P. synxantha* BG33R, cloned in shuttle vector pJB3Tc20, was introduced in PmnH-susceptible strain LMG 1794 and conveyed full immunity in a spot assay with PmnH (and with its H224A and D227A mutants) (Fig. 3E), indicative of protection against ColN domain-mediated inhibition. Careful inspection of the context of *pmnH* did not readily reveal a second candidate immunity partner, unlike putative bacteriocins PsdH1 and PsdH2, which are accompanied by two putative immunity modules downstream (3).

### PmnH and PseuM bacteriocins target the ferrichrome transducer.

**FIG 4:**
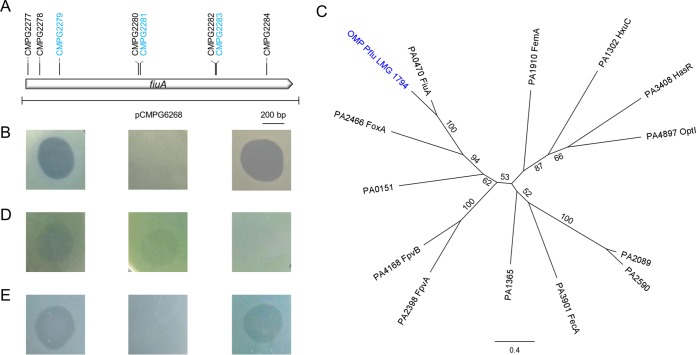
(A) Schematic representation of the targeted LMG 1794 gene with plasposon insertion sites generating mutants with absent PmnH-susceptible phenotype. Complemented mutants are colored blue. (B) Spot assay of PmnH against LMG 1794 wild type (left), mutant CMPG2281 carrying empty control vector pJB3Tc20 (middle), and CMPG2281 with pCMPG6268 (complementation, right). (C) ML phylogenetic tree of the STN and plug domains of TonB-dependent transporters from *Pseudomonas aeruginosa* PAO1 and the PmnH-targeted OMP from *P. fluorescens* LMG 1794 (colored blue). The scale bar represents 0.4 substitutions per site. Bootstrap values (percentages of 1,000 replicates) higher than 50 are shown at the branches. (D) Spot assay of PseuM_NCTC10332_ against *P. aeruginosa* PAO1 wild type (left), *fecA* mutant (PA3901, middle), and *fiuA* mutant (PA0470, right). (E) Spot assay of PseuM_Q8r1-96_ against *P. fluorescens* F113 wild type (left), F113 *fiuA-*deletion mutant CMPG2285 (middle), and CMPG2285 complemented with *fiuA*_F113_ (right).

A transposon mutagenesis approach was pursued in order to identify the cell surface target of PmnH, using indicator strain *P. fluorescens* LMG 1794. Transconjugants were pooled, plated, and exposed to spots of recombinant PmnH. Colonies growing inside halos were selected. After confirmation of the resistance phenotype, flanking sequences of 11 mutants were determined. Of these, eight unique mutants carried the mobile element inserted in the same gene, predicted to encode an OMP (Fig. 4A). The involvement of the targeted OMP in conferring PmnH susceptibility was confirmed via complementation with the wild-type gene from strain *P. fluorescens* LMG 1794. Three different transposon insertion mutants examined (CMPG2279, CMPG2281, and CMPG2283) hereby regained their original bacteriocin susceptibility (Fig. 4B).

The motifs and domains present in the affected LMG 1794 gene product comprise a Sec-dependent signal peptide, a “secretin and TonB N terminus short domain” (STN; PF07660), a plug domain (PF07715), and a TonB-dependent receptor domain (PF00593) (37, 38). The STN domain is required for interaction with regulatory proteins and subsequent sigma factor activation, as part of cell surface signaling systems. As such, this amino-terminal extension discriminates TonB-dependent transducers from TonB-dependent receptors that lack an STN domain (39). The pseudomonad TonB-dependent outer membrane proteins have been extensively studied, in particular in *P. aeruginosa* (40–43), although the function of several of its predicted transporters remains elusive. Comparison of the identified OMP to 13 TonB-dependent transporters with an STN domain present in strain PAO1 revealed striking homology to FiuA (62.1% pairwise amino acid identity). FiuA accounts for the uptake of the majority of iron-bound ferrichrome by *P. aeruginosa* (44, 45). The next best sequence match among the PAO1 transporters was that to the ferrioxamine-responsive FoxA (only 34.7% amino acid identity). The close similarity of the LMG 1794 OMP to FiuA is illustrated in the phylogenetic tree constructed from alignment of the region comprising the STN and plug domains that is well conserved among the transporters (Fig. 4C). The presence in strain LMG 1794 of a syntenic tripartite module encoding homologues of the extracytoplasmic function (ECF) sigma factor (PA0472; 74% amino acid identity) and anti-sigma factor (PA0471; 51% amino acid identity) functionally linked to PAO1 FiuA (PA0470) further supports the assignment of the LMG 1794 target as a ferrichrome-binding OMP. Interestingly, the ferrichrome receptor in *E. coli* (FhuA) was previously found to be the outer membrane target of colicin M (46).

In light of (i) the structural similarities observed between the amino-terminal domains of PseuMs and colicin M and (ii) the (low) homology between the amino-terminal domains of PmnH and previously characterized PseuMs from *P. aeruginosa* NCTC10332, *P. fluorescens* Q8r1-96, and *P. syringae* pv. tomato DC3000 (Fig. S3), we examined the possibility that FiuA may be parasitized by PseuM bacteriocins as well. To this end, a *fiuA* transposon mutant of *P. aeruginosa* PAO1 (PA0470) (47) was tested for altered susceptibility to the PseuM bacteriocin from *P. aeruginosa* NCTC10332. Previously, it was found that strain PAO1 displays a turbid-halo phenotype for this PseuM bacteriocin (16). No such halo could be observed for this *fiuA* mutant, whereas a *fecA* mutant, mutated in another TonB-dependent iron uptake transporter, still displays the wild-type phenotype (Fig. 4D). Furthermore, a mutant with a deleted *fiuA* homologue (PSF113_RS57875) was constructed for *P. fluorescens* F113, a strain susceptible to the PseuM bacteriocin from *P. fluorescens* Q8r1-96 (16). This mutant proved insensitive to PseuM_Q8r1-96_, and susceptibility was restored via complementation (Fig. 4E).

Taken together, these results provide evidence that ColM-like *Pseudomonas* bacteriocins indeed target FiuA, hereby supporting the hypothesis that the structural similarity between PseuM and colicin M points to a shared functional ancestor (13). The substantial sequence divergence among FiuA homologues from those strains of the *P. fluorescens* group that were included in our test panel (79.1% average pairwise amino acid identity, 55.4% identical sites) may explain why not all *P. fluorescens* strains are susceptible. Notably, FiuA sequences of PmnH-susceptible *P. fluorescens* strains tend to cluster closely (Fig. S5).

10.1128/mBio.01961-16.5 FIG S5  Maximum likelihood phylogenetic tree of ferrichrome transducer homologues from strains assigned to the *P. fluorescens* group that were used in this study and for which genome sequence information is available. The scale bar represents 0.08 substitutions per site. Bootstrap values (percentages of 1,000 replicates) are shown at the branches. Proteins originating from strains that are susceptible to PmnH are colored blue. Species abbreviations are the same as in Fig. 1. Additional abbreviations: Psim, *Pseudomonas simiae*; Ppro, *Pseudomonas protegens*. Download FIG S5, TIF file, 0.2 MB. Copyright © 2017 Ghequire et al. 2017 Ghequire et al. This content is distributed under the terms of the Creative Commons Attribution 4.0 International license.

## DISCUSSION

By examining the functionality of a putative *Pseudomonas* bacteriocin with an unprecedented dual ColM-ColN domain architecture, PmnH, we could attribute killing activity to its ColN-type pore-forming module, identify the membrane protein ImnH as a ColN immunity-providing partner, and show that for entry into susceptible cells the ferrichrome transporter is parasitized, similarly to mono-ColM-domain bacteriocins of pseudomonads.

In addition to the pyocin S5 bacteriotoxin domain of *P. aeruginosa*, phylogenetically related to the enterobacterial colicin Ia, this study identifies a second type of pore-forming activity in pseudomonads, sharing apparent ancestry with the N-type colicin. Whether the additional domain of PmnH, exhibiting striking similarity to ColM-type bacteriocins from enterobacteria and pseudomonads, contributes to its functionality, possibly broadening its target reach, could not be resolved since a strain susceptible to a partially disabled PmnH, stripped of its pore-forming domain, could not yet be identified. Somewhat arguing against enzymatic functionality of the ColM-like domain are (i) the diverged sequences at the presumed active site for most strains (even more degenerate in the PmnH sequence from Pseudomonas sp. 25R14), and (ii) the lack of a second linked immunity gene. It should be pointed out that deviations from the prototypical catalytic ColM motif do not necessarily preclude bacteriocin functionality (30). In line with this, it was recently found that the pectobacterial ColM-type bacteriocin pectocin M1 and a mutant form with a modified catalytic motif both provoke cellular lysis when expressed in the periplasm of *E. coli* (48). By demonstrating that only the pectocin M1 wild type retains lipid II degradation activity, this study indicates that toxicity exerted by the ColM module may be more complex than merely enzymatic, as initially thought. Consequently, a strictly conserved catalytic motif in PmnH’s ColM module may not be essential to interfere with the peptidoglycan metabolism.

In view of the respective immunity pairs accompanying the predicted hybrid nuclease bacteriocins PsdH1 and PsdH2 (3), the presence of a single immunity gene linked to a dual-domain bacteriocin is rather unexpected. One cannot entirely exclude that an immunity protein with relaxed protective capacity, linked to a bacteriocin of the same type but encoded elsewhere in the genome, or encoded by a nonlinked immunity gene, might prevent self-intoxication. The latter possibility has been proposed for the ColM-type pectocin from *Pectobacterium carotovorum* PBR1692 and its putative *cmi-*like immunity gene, separated by at least 84 kb (49). Alternatively, since divergence among (putative) ColN-type immunity proteins in pseudomonads is considerable (only 30.5% pairwise amino acid identity), one might speculate that membrane protein ImnH may provide dual protection by expanded immunity capacity. This would be reminiscent of PmiA membrane proteins that are promiscuous in providing protection against multiple ColM-like PseuM bacteriocins (16). On the other hand, if PmnH’s ColM domain interferes nonenzymatically with peptidoglycan metabolism, it is conceivable that a single membrane-associated protein may concomitantly provide protection against two bacteriocin activities that act on neighboring cell envelope constituents.

The targeting of the FiuA ferrichrome transporter by PmnH and PseuM bacteriocins is well in line with the previous observations on S-type pyocins aiming at TonB-dependent OMPs that are involved in iron uptake and supports the hypothesis of shared ancestry of ColM-type *Pseudomonas* bacteriocins and colicin M from *E. coli*. Besides parasitism of determinants involved in the “fight-for-iron” by modular bacteriocins from *Pseudomonas* (50), a similar strategy is adopted by a number of bacteriocins, such as colicins (e.g., ferric enterobactin receptor FepA by colicin B) (51), FhuA by microcin J25 (52), FusA by pectocin M1 (53), and yersiniabactin receptor FyuA by pesticin (54), underlining the pivotal role of iron for bacterial survival. At present, it is not obvious which evolutionary scenario generated *pmnH*: a ColM-type bacteriocin gene that recruited a *colN*/*immN* module or an N-type bacteriocin gene into which a ColM-type bacteriocin gene became integrated.

Modular bacteriocins constitute an attractive novel drug lead in combating a number of persistent infections caused by Gram-negative bacteria. Schulz et al. (55) recently demonstrated that *in planta*-produced colicins can successfully clear *E. coli-*spiked meat, and the potential of (several) pyocins in eradicating *P. aeruginosa* in a murine lung model has equally been evidenced (56). A major pitfall, however, in the use of bacteriocins, especially for the treatment of *P. aeruginosa*, is the presence of orphan immunity genes. Therefore, preference should be given to the design of engineered pyocins with frequently occurring targets coupled to toxin-immunity modules that are less common in *Pseudomonas* genomes and for which orphan immunity genes are rare. The picture of bacteriocin susceptibility is made more complex by the occurrence of additional immunity genes downstream of certain bacteriocin-immunity gene pairs (3), possibly serving as a reservoir to trap invading pyocins, and by immunity proteins displaying a relaxed immunity phenotype. Instead of acquisition of an entire toxin-immunity module with different functionalities to deal with opponents armored with immunity against a particular bacteriocin, the recruitment of a second toxin module to generate modular bacteriocins with dual toxin organization may therefore represent a cunning bacterial strategy to circumvent bacteriocin resistance. In this context, the combination of several toxin modules in one pyocin may also reduce the complexity of bacteriocin cocktails and deserves further scrutiny as a tool to combat *P. aeruginosa*.

## MATERIALS AND METHODS

### Strains and growth conditions.

Bacterial strains used in this study are listed in Table S2 in the supplemental material. *Pseudomonas* strains were grown in Casamino Acids medium, King’s B medium, or tryptic soy broth (TSB), and *Escherichia coli* was grown in 2.5% LB (media were from BD Bacto and MP Biomedicals). *E. coli* and *P. aeruginosa* were grown at 37°C, and other *Pseudomonas* strains were grown at 30°C with shaking at 200 rpm. Plasmids were propagated in *E. coli* TOP10F′ (Invitrogen), and *E. coli* Rosetta (DE3)pLysS (Novagen) was used for production of recombinant proteins. Growth media were supplemented with filter-sterilized antibiotics when needed: ampicillin (100 µg/ml; Sigma-Aldrich), chloramphenicol (15 µg/ml; Sigma-Aldrich), kanamycin (50 µg/ml; Sigma-Aldrich), and tetracycline (12.5 µg/ml; Sigma-Aldrich). Media were solidified with bacteriological agar (1.5%; VWR). Bacterial strain stocks were stored at −80°C in 25% (vol/vol) glycerol.

10.1128/mBio.01961-16.7 TABLE S2  Strains and plasmids used in this study. Download TABLE S2, DOCX file, 0.02 MB. Copyright © 2017 Ghequire et al. 2017 Ghequire et al. This content is distributed under the terms of the Creative Commons Attribution 4.0 International license.

### Plasmid construction.

Genomic DNA was isolated with the Puregene Yeast/Bact. kit B (Qiagen). Plasmids were harvested with the GenElute HP Plasmid Miniprep kit (Sigma-Aldrich). Competent *E. coli* cells for heat shock transformation were prepared using standard methods (57), and electrocompetent *Pseudomonas* cells were prepared with sucrose (VWR) (58). Restriction enzymes (New England BioLabs) were used as specified by the supplier, and DNA fragments were ligated with T4 DNA ligase (Invitrogen). Plasmid constructs were sequenced for validation by GATC Biotech (Constance, Germany). PCR amplicons were generated with Q5 high-fidelity DNA polymerase (New England BioLabs). Genomic DNA was used as a template, and primers are listed in Table S3. Standard procedures were followed for DNA electrophoresis. PCR fragments were purified with the GenElute PCR cleanup kit (Sigma-Aldrich), digested, and ligated in pET28a, pJB3Tc20, or pAKE604. Point mutant constructs encoding truncated PmnH forms were generated via splicing by overlap extension. Plasmids used in this study are summarized in Table S2.

10.1128/mBio.01961-16.8 TABLE S3  Primers used in this study. Download TABLE S3, DOCX file, 0.02 MB. Copyright © 2017 Ghequire et al. 2017 Ghequire et al. This content is distributed under the terms of the Creative Commons Attribution 4.0 International license.

### Overexpression and purification of recombinant bacteriocins.

Following heat shock transformation to *E. coli* Rosetta (DE3)pLysS, bacteria containing the plasmid of interest were grown in 500-ml Erlenmeyer flasks until the optical density at 600 nm (OD_600_) reached 0.7. Next, cultures were induced with isopropyl-β-d-1-thiogalactopyranoside (Formedium) at a 1 mM final concentration and incubated for 16 h at 20°C with shaking at 200 rpm. Next day, cells were harvested (5,000 × *g*, 20 min), and pellets were frozen overnight. Subsequently, cells were thawed, resuspended in lysis buffer (300 mM NaCl [VWR], 50 mM NaH_2_PO_4_ [VWR], 10 mM imidazole [Sigma-Aldrich], pH 8.0), and sonicated with a Branson Digital Sonifier 250 (18%, 10 cycles of 30 s on and 30 s off). Next, extracts were supplemented with nuclease (0.01 U/µl; Sigma-Aldrich), incubated at 37°C for 0.5 h, centrifuged (10,000 × *g*, 30 min), and filtered (0.20 µm pore size; Sarstedt). Soluble proteins were subsequently mounted on a 5-ml His-trap column (GE Healthcare), and His-tagged proteins were eluted with a linear gradient of 10 to 500 mM imidazole. The presence of recombinant protein was verified on Coomassie blue-stained SDS-PAGE gels. Elution fractions were pooled, concentrated with Vivaspin filters (10,000 Da; Sartorius), and polished via gel filtration with a Superdex 200 column 16/60 (GE Healthcare), using Tris buffer (20 mM Tris [Sigma-Aldrich], 200 mM NaCl, pH 7.5) as running buffer.

### Bacteriocin assay.

Activity of recombinant His-tagged protein was tested via spot assay. Ten-microliter drops of protein were put on top of a lawn of bacteria. After drying, plates were incubated overnight. On the following day, bacteriocin activity was detected as a halo appearing in the cell lawn. Dialysis buffer was used as a control. The role of iron was tested via addition of FeCl_3_ (50 µM; Sigma-Aldrich) to CAA.

### Plasposon mutagenesis and isolation of bacteriocin-resistant indicator mutants.

A plasposon mutant library of *P. fluorescens* LMG 1794 was generated via biparental conjugation using *E. coli* BW20767 carrying transposon delivery vector pRL27 as a donor. Transconjugants were selected on tryptic soy agar (TSA) supplemented with kanamycin and grown at 16°C for 72 h. Random insertion of the transposon was verified by randomly selecting 10 LMG 1794 mutants following the plasposon rescue procedure and sequencing the flanking insertion positions (primers listed in Table S3). To isolate PmnH-resistant mutants, all *P. fluorescens* LMG 1794 plasposon mutants were pooled and 10-fold dilutions were plated on TSA supplemented with kanamycin. Afterward, 50-µl spots of purified PmnH at high concentrations were added. On the following day, resistant clones could be retrieved as colonies growing inside halos. After being streaked to single colonies, the resistance phenotype of the mutants was confirmed, and the insertion site was determined.

### Construction of a *P. fluorescens* F113 *fiuA* mutant.

A deletion mutant in the *fiuA* homologue of *P. fluorescens* F113 (PSF113_RS57875) was constructed by ligating ~500-bp fragments upstream and downstream of *fiuA* in suicide plasmid pAKE604. Sequence-verified plasmid was transformed to *E. coli* S17-1 and transferred to *P. fluorescens* F113 by biparental conjugation. F113 conjugants were streaked to single colonies in the presence of kanamycin and individually grown in a test tube without antibiotic for 12 h. Next, 10-fold serial dilutions were plated on TSA containing sucrose (8%; VWR) to select for the loss of the cassette, and allelic exchange of the recombinants was validated by PCR using *Taq* polymerase (New England BioLabs), using F113-specific primers (Table S3). As expected, in about half of the randomly selected mutants *fiuA* was knocked out. The deletion construct resulted in a start-stop fusion with an interspersed Gly-Ser (originating from the fragment-fusing BamHI site) pair and was validated via PCR amplification and subsequent sequencing. The construction of the F113 *fiuA* mutant was executed in parallel to obtain four independent F113 mutant strains.

### Phylogenetic and gene synteny analysis.

Homology searches were performed via Blast searches, using the NCBI nonredundant database. Sequence alignments and phylogenetic analyses were performed with MUSCLE and PhyML, respectively, implemented in Geneious 7.1.7 (Biomatters Ltd., Auckland, New Zealand). Domain analyses were conducted with SMART (http://smart.embl-heidelberg.de) and InterPro (https://www.ebi.ac.uk/interpro). The progressive MAUVE algorithm, implemented in Geneious 7.1.7, was used to explore gene synteny (59).

### Accession number.

 A draft genome sequence of *P. fluorescens* DSM 50090 (LMG 1794) has been released under a whole-genome sequencing (WGS) project of *P. fluorescens* DSM 50090 (LHVP01000000) (60).
