# Supplementary figures and images for: A Natural Chimeric Pseudomonas Bacteriocin with Novel Pore-Forming Activity Parasitizes the Ferrichrome Transporter
Source: mBio. 2017 Feb 21;8(1):e01961-16. doi: 10.1128/mBio.01961-16 (PMC5358913; doi:10.1128/mBio.01961-16)

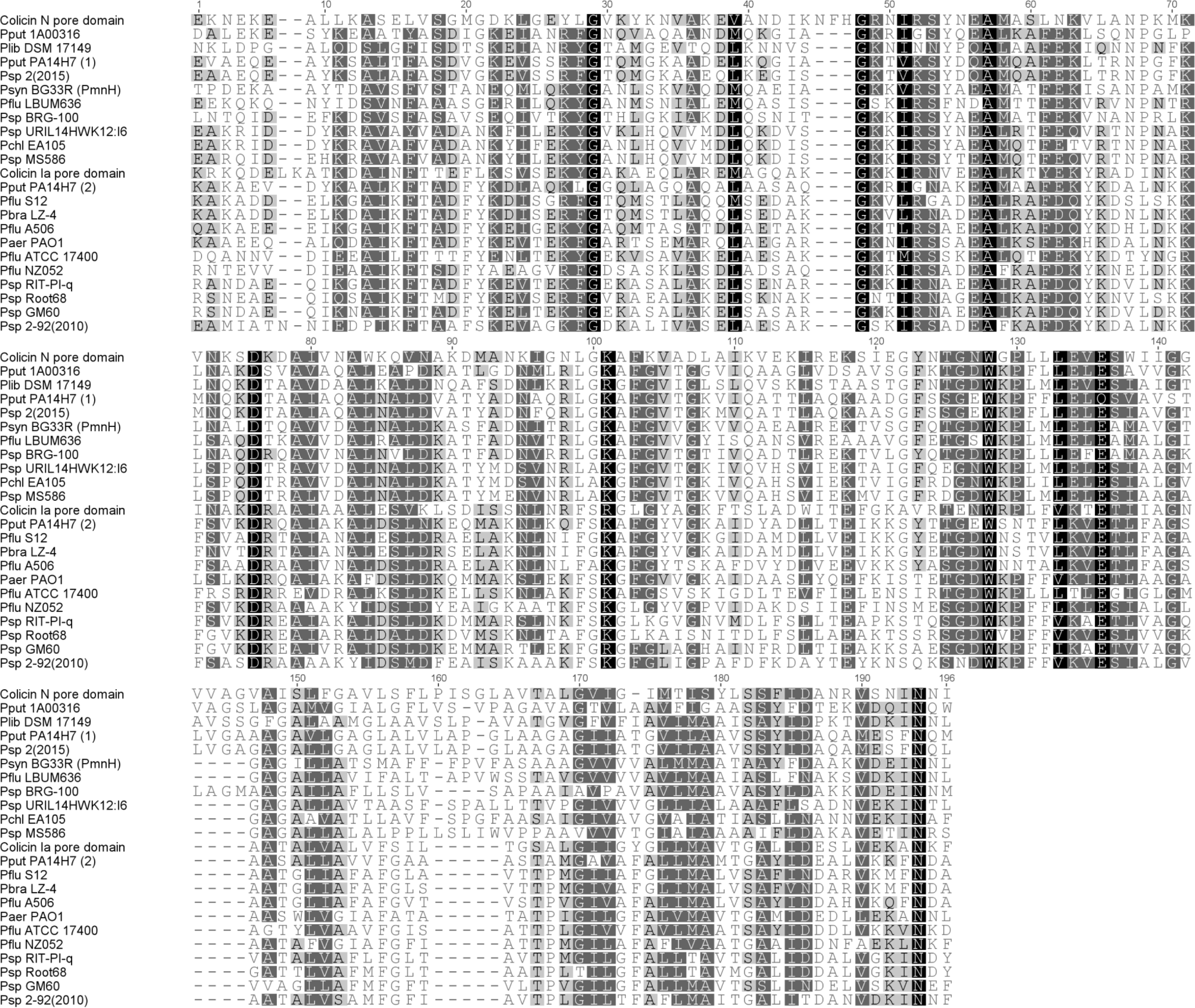

Supplement: FIG S1 [file mbo001173196sf1.tif]

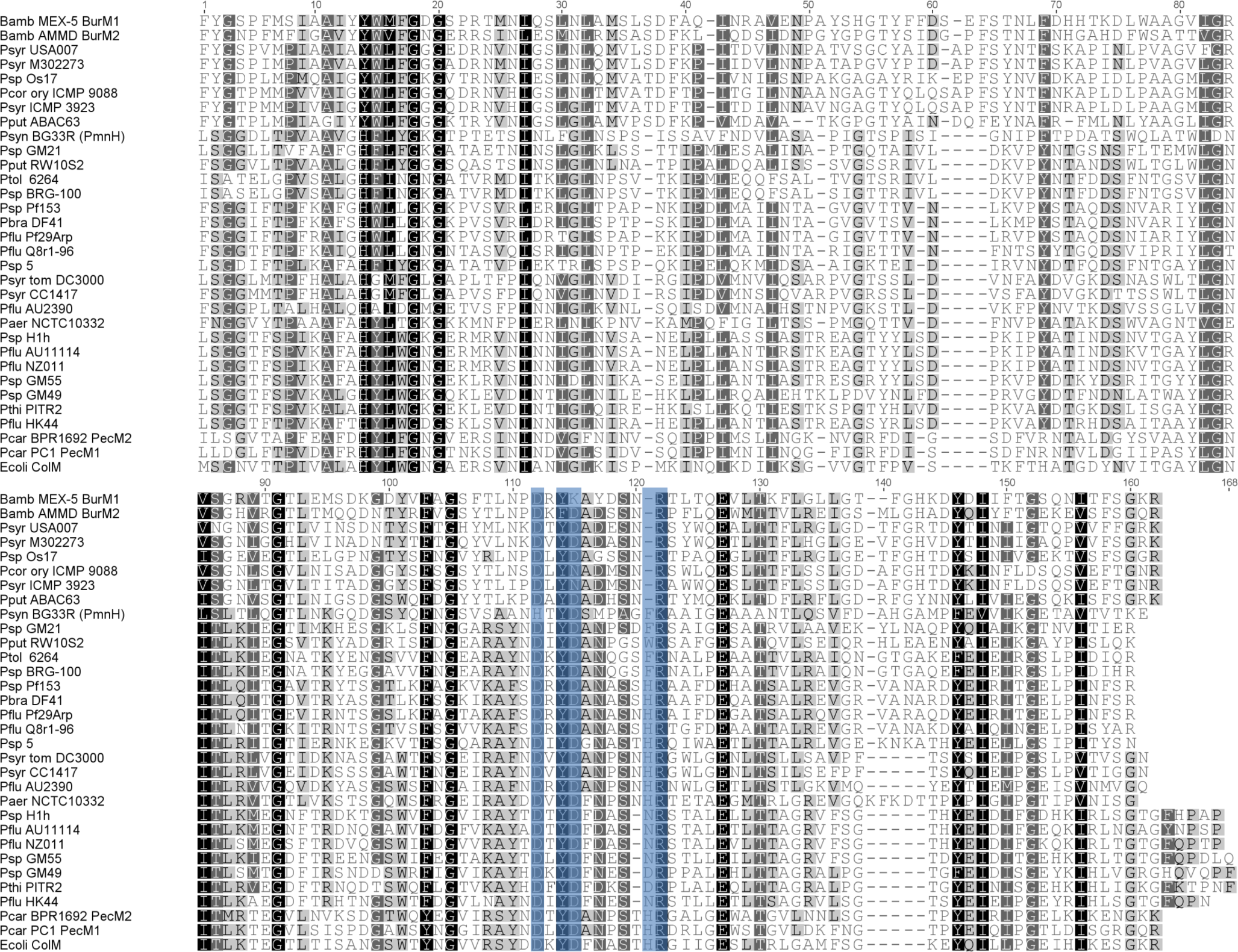

Supplement: FIG S2 [file mbo001173196sf2.tif]

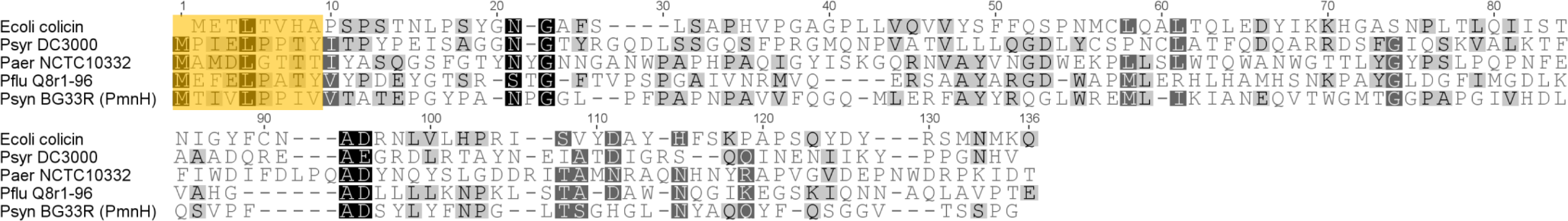

Supplement: FIG S3 [file mbo001173196sf3.tif]

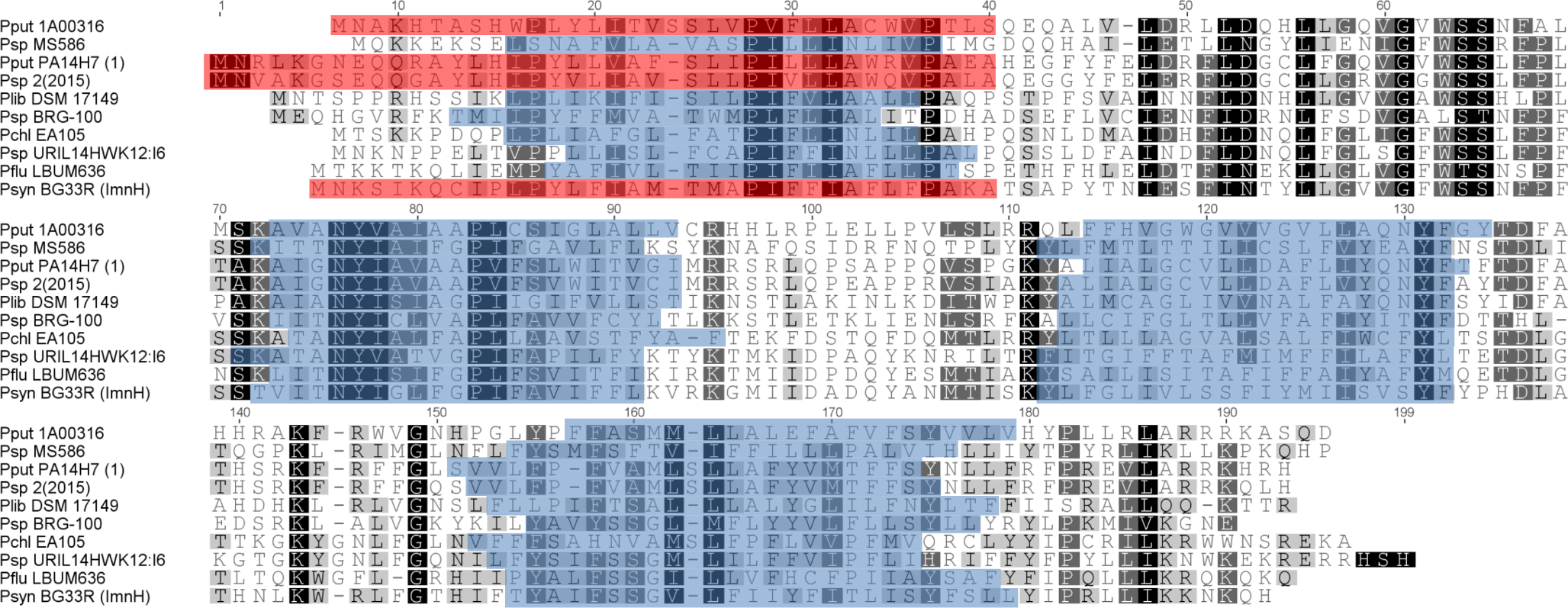

Supplement: FIG S4 [file mbo001173196sf4.tif]

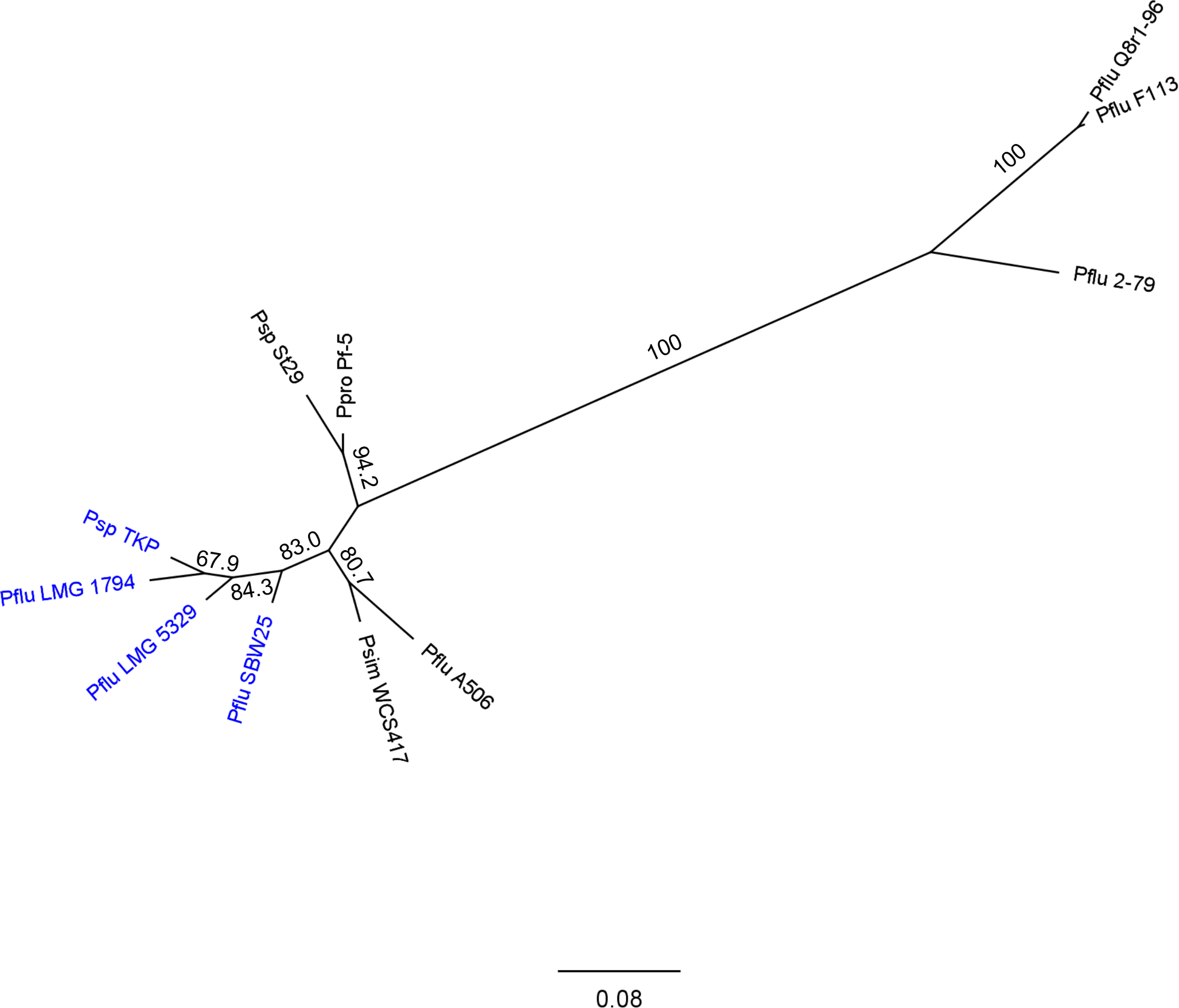

Supplement: FIG S5 [file mbo001173196sf5.tif]
