# Supplementary material for: A Natural Chimeric Pseudomonas Bacteriocin with Novel Pore-Forming Activity Parasitizes the Ferrichrome Transporter
Source: mBio. 2017 Feb 21;8(1):e01961-16. doi: 10.1128/mBio.01961-16 (PMC5358913; doi:10.1128/mBio.01961-16)
Supplement: TABLE S1 [file mbo001173196st1.docx]

Table S1. Antibacterial activity of purified recombinant PmnH against *Pseudomonas* spp.: +, susceptible (clear halo); T, turbid (semi-transparent halo); -, no halo.

| **Strain** | **PmnH susceptibility** |
| --- | --- |
| *P. aeruginosa* PAO1 | - |
| *P. aeruginosa* PA14 | - |
| *P. chlororaphis* subsp. *aureofaciens* LMG 1245 | - |
| *P. cichorii* LMG 2162 | - |
| *P. entomophila* L48 | - |
| *P. fluorescens* 2-79 | - |
| *P. fluorescens* 13-79 | - |
| *P. fluorescens* A506 | - |
| *P. fluorescens* F113 | - |
| *P. fluorescens* LMG 1794 | + |
| *P. fluorescens* LMG 5329 | + |
| *P. fluorescens* Q8r1-96 | - |
| *P. fluorescens* SBW25 | T |
| *P. fluorescens* VA 1.2 | - |
| *P. fluorescens* VA 2.1 | - |
| *P. fluorescens* WCS365 | - |
| *P. protegens* Pf-5 | - |
| *P. putida* W619 | - |
| *P. putida* WCS358 | - |
| *P. putida* GB-1 | - |
| *P. putida* LMG 2257 | - |
| *P. putida* KT2440 | - |
| *P. putida* OE 47.1 | - |
| *P. putida* OE 53.2 | - |
| *P. resinovorans* LMG 2274 | - |
| *P. savastanoi* pv. glycinea LMG 5066 | - |
| *P. savastanoi* pv. *savastanoi* LMG 2209 | - |
| *P. simiae* WCS417 | - |
| *P. stutzeri* LMG 11199 | - |
| *P. syringae* GR12-2R3 | - |
| *P. syringae* pv. *syringae* LMG 1247 | - |
| *P. syringae* pv. *tabaci* LMG 5192 | - |
| *P. syringae* pv. *tomato* DC3000 | - |
| *Pseudomonas* sp. St29 | - |
| *Pseudomonas* sp. TKP | + |
