# Supplementary material for: A Natural Chimeric Pseudomonas Bacteriocin with Novel Pore-Forming Activity Parasitizes the Ferrichrome Transporter
Source: mBio. 2017 Feb 21;8(1):e01961-16. doi: 10.1128/mBio.01961-16 (PMC5358913; doi:10.1128/mBio.01961-16)
Supplement: TABLE S2 [file mbo001173196st2.docx]

Table S2. Strains and plasmids used in this study.

| **Plasmid/strain** | **Purpose of use/genotype/characteristics** | **Reference or source** |
| --- | --- | --- |
| pET28a | pBR322 origin, His-tag/thrombin/T7 tag; Km^R^ | Novagen |
| pJB3Tc20 | Broad-host-range cloning vector; Ap^R^, Tc^R^ | (1) |
| pAKE604 | Suicide plasmid, *oriT lacZ sacB*; Ap^R^ Km^R^ | (2) |
| pRL27 | Tn*5*-RL27 (Km^R^-*ori*R6 K) delivery vector | (3) |
| pCMPG6238 | pET28a with 1385-bp PCR-amplified fragment containing *pmnH* from *P. synxantha* BG33R, cloned in NcoI/XhoI | This study |
| pCMPG6249 | pET28a with 818-bp PCR-amplified fragment containing *pmnH∆colN* from *P. synxantha* BG33R, cloned in NcoI/XhoI | This study |
| pCMPG6250 | pET28a containing ColM-like bacteriocin gene from *P. aeruginosa* NCTC10332 | (4) |
| pCMPG6253 | pJB3Tc20 with 587-bp PCR-amplified fragment containing *imnH* from *P. synxantha* BG33R, cloned in PstI/EcoRI | This study |
| pCMPG6262 | Mutant construct of pCMPG6238; H224A of the encoded PmnH | This study |
| pCMPG6263 | Mutant construct of pCMPG6238; D227A of the encoded PmnH | This study |
| pCMPG6268 | pJB3Tc20 with 2526-bp PCR-amplified fragment containing targeted *omp* from *P. fluorescens* LMG 1794, cloned in HindIII/BamHI | This study |
| pCMPG6271 | pET28a containing ColM-like bacteriocin gene from *P. fluorescens* Q8r1-96 | (4) |
| pCMPG6278 | pAKE604 containing 547-bp and 546-bp PCR-amplified fragments upstream and downstream, respectively, from *fiuA* from *P. fluorescens* F113, cloned in HindIII/EcoRI | This study |
| pCMPG6279 | pJB3Tc20 with 2500-bp PCR-amplified fragment containing *fiuA* from *P. fluorescens* F113, cloned in HindIII/BamHI | This study |
|  |  |  |
| TOP10F’ | F’[*lac*I^q^ Tn*10*(Tet^R^)] *mcr*A ∆(*mrr-hsd*RMS-*mcr*BC) Φ80*lac*Z∆M15 ∆*lac*X74 *rec*A1 *ara*D139 ∆(*ara-leu*)7697 *gal*U *gal*K *rps*L *end*A1 *nup*G | Invitrogen |
| BW20767 | *E. coli* donor of pRL27 | (3) |
| S17-1 | *rec*A *pro hsd*R RP4-2-Tc::Mu:Km::Tn*7* | CMPG collection |
| Rosetta (DE3)pLysS | F^-^ *omp*T *hsd*S_B_(r_B_^-^ *m_B_*^-^) *gal dcm* (DE3) pLysSRARE (Cam^R^) | Novagen |
|  |  |  |
| CMPG2277 | PmnH-resistant LMG 1794 mutant, hit in *fiuA* | This study |
| CMPG2278 | PmnH-resistant LMG 1794 mutant, hit in *fiuA* | This study |
| CMPG2279 | PmnH-resistant LMG 1794 mutant, hit in *fiuA* | This study |
| CMPG2280 | PmnH-resistant LMG 1794 mutant, hit in *fiuA* | This study |
| CMPG2281 | PmnH-resistant LMG 1794 mutant, hit in *fiuA* | This study |
| CMPG2282 | PmnH-resistant LMG 1794 mutant, hit in *fiuA* | This study |
| CMPG2283 | PmnH-resistant LMG 1794 mutant, hit in *fiuA* | This study |
| CMPG2284 | PmnH-resistant LMG 1794 mutant, hit in *fiuA* | This study |
| CMPG2285 | *P. fluorescens* F113 *fiuA* deletion mutant | This study |
| PA0470 | Transposon mutant of *P. aeruginosa* PAO1, hit in *fiuA* | (5) |
| PA3901 | Transposon mutant of *P. aeruginosa* PAO1, hit in *fecA* | (5) |
|  |  |  |
| *P. aeruginosa* PAO1 | Opportunistic pathogen, human isolate | (6) |
| *P. aeruginosa* PA14 | Opportunistic pathogen, human isolate | (6) |
| *P. chlororaphis* subsp. *aureofaciens* LMG 1245 | Maas River clay suspended in kerosene | BCCM/LMG collection |
| *P. cichorii* LMG 2162 | *Cichorium endivia* | BCCM/LMG collection |
| *P. entomophila* L48 | Entomopathogenic strain that kills *Drosophila melanogaster* | (6) |
| *P. fluorescens* 2-79 | Wheat rhizosphere | (6) |
| *P. fluorescens* 13-79 | Wheat rhizosphere | (7) |
| *P. fluorescens* A506 | Pear isolate | (6) |
| *P. fluorescens* F113 | Sugarbeet rhizosphere | (6) |
| *P. fluorescens* LMG 1794 | Pre-filter water-works tanks | BCCM/LMG collection |
| *P. fluorescens* LMG 5329 | Cultivated mushroom | BCCM/LMG collection |
| *P. fluorescens* Q8r1-96 | Wheat rhizosphere | (8) |
| *P. fluorescens* SBW25 | Sugarbeet leaves | (6) |
| *P. fluorescens* VA 1.2 | Rhizosphere wheat | CMPG collection |
| *P. fluorescens* VA 2.1 | Caulosphere wheat | CMPG collection |
| *P. fluorescens* WCS365 | Potato rhizosphere | (9) |
| *P. protegens* Pf-5 | Soil | (6) |
| *P. putida* W619 | *Populus trichocarpa* | (6) |
| *P. putida* WCS358 | Potato rhizosphere | (6) |
| *P. putida* GB-1 | Fresh water isolate, manganese oxidizer | (6) |
| *P. putida* LMG 2257 | Soil by lactate enrichment | BCCM/LMG collection |
| *P. putida* KT2440 | Plasmid-free derivative of toluene-degrading isolate *P. putida* mt-2, rhizosphere colonizer | (6) |
| *P. putida* OE 47.1 | Maize rhizosphere | CMPG collection |
| *P. putida* OE 53.2 | Maize rhizosphere | CMPG collection |
| *P. resinovorans* LMG 2274 | Soil | BCCM/LMG collection |
| *P. savastanoi* pv. glycinea LMG 5066 | *Glycine max* cv. Disoy, affected by leaf blight | BCCM/LMG collection |
| *P. savastanoi* pv. *savastanoi* LMG 2209 | *Olea europaea* | BCCM/LMG collection |
| *P. simiae* WCS417 | Wheat rhizosphere | (6) |
| *P. stutzeri* LMG 11199 | Spinal fluid | BCCM/LMG collection |
| *P. syringae* GR12-2R3 | Grass rhizosphere | (10) |
| *P. syringae* pv. *syringae* LMG 1247 | *Syringa vulgaris* | BCCM/LMG collection |
| *P. syringae* pv. *tabaci* LMG 5192 | *Nicotiana tabacum* | BCCM/LMG collection |
| *P. syringae* pv. *tomato* DC3000 | Bacterial speck on tomato and *Arabidopsis* | (6) |
| *Pseudomonas* sp. St29 | Potato rhizosphere | (6) |
| *Pseudomonas* sp. TKP | Non-γ-hexachlorocyclohexane-degrader | (6) |

**References**

1. **Blatny JM, Brautaset T, Winther-Larsen HC, Haugan K, Valla S.** 1997. Construction and use of a versatile set of broad-host-range cloning and expression vectors based on the RK2 replicon. Appl Environ Microbiol **63:**370-379.

2. **El-Sayed AK, Hothersall J, Thomas CM.** 2001. Quorum-sensing-dependent regulation of biosynthesis of the polyketide antibiotic mupirocin in Pseudomonas fluorescens NCIMB 10586. Microbiology **147:**2127-2139. 10.1099/00221287-147-8-2127

3. **Larsen RA, Wilson MM, Guss AM, Metcalf WW.** 2002. Genetic analysis of pigment biosynthesis in Xanthobacter autotrophicus Py2 using a new, highly efficient transposon mutagenesis system that is functional in a wide variety of bacteria. Arch Microbiol **178:**193-201. 10.1007/s00203-002-0442-2.

4. **Ghequire MG, Kemland L, De Mot R.** 2017. Novel immunity proteins associated with colicin M-like bacteriocins exhibit promiscuous protection in *Pseudomonas*. Front Microbiol **8:**93. 10.3389/fmicb.2017.00093.

5. **Jacobs MA, Alwood A, Thaipisuttikul I, Spencer D, Haugen E, Ernst S, Will O, Kaul R, Raymond C, Levy R, Chun-Rong L, Guenthner D, Bovee D, Olson MV, Manoil C.** 2003. Comprehensive transposon mutant library of *Pseudomonas aeruginosa*. Proc Natl Acad Sci U S A **100:**14339-14344. 10.1073/pnas.2036282100.

6. **Winsor GL, Griffiths EJ, Lo R, Dhillon BK, Shay JA, Brinkman FS.** 2016. Enhanced annotations and features for comparing thousands of *Pseudomonas* genomes in the *Pseudomonas* genome database. Nucleic Acids Res **44:**D646-653. 10.1093/nar/gkv1227.

7. **Weller DM, Cook RJ.** 1983. Suppression of Take-all of wheat by seed treatments with fluorescent pseudomonads. Phytopathology **73:**463-469.

8. **Loper JE, Hassan KA, Mavrodi DV, Davis EW, 2nd, Lim CK, Shaffer BT, Elbourne LD, Stockwell VO, Hartney SL, Breakwell K, Henkels MD, Tetu SG, Rangel LI, Kidarsa TA, Wilson NL, van de Mortel JE, Song C, Blumhagen R, Radune D, Hostetler JB, Brinkac LM, Durkin AS, Kluepfel DA, Wechter WP, Anderson AJ, Kim YC, Pierson LS, 3rd, Pierson EA, Lindow SE, Kobayashi DY, Raaijmakers JM, Weller DM, Thomashow LS, Allen AE, Paulsen IT.** 2012. Comparative genomics of plant-associated Pseudomonas spp.: insights into diversity and inheritance of traits involved in multitrophic interactions. PLoS Genet **8:**e1002784. 10.1371/journal.pgen.1002784.

9. **Geels FP, Schippers B.** 1983. Selection of antagonistic fluorescent *Pseudomonas* spp. and their root colonization and persistence following treatment of seed potatoes. Journal of Phytopathology **108:**193-206. 10.1111/j.1439-0434.1983.tb00579.x.

10. **Lifshitz RJ, Kloepper JW, Kozlowski M, Simonson C, Carlson J, Tipping EM, Zaleska I.** 1987. Growth promotion of canola (rapeseed) seedlings by a strain of *Pseudomonas putida* under gnotobiotic conditions. Canadian Journal of Microbiology **33:**390-395. 10.1139/m87-068.
