# Supplementary material for: A Natural Chimeric Pseudomonas Bacteriocin with Novel Pore-Forming Activity Parasitizes the Ferrichrome Transporter
Source: mBio. 2017 Feb 21;8(1):e01961-16. doi: 10.1128/mBio.01961-16 (PMC5358913; doi:10.1128/mBio.01961-16)
Supplement: TABLE S3 [file mbo001173196st3.docx]

Table S3. Primers used in this study.

| **Number** | **Sequence^a^ (5’-3’)** | **Purpose of use** |
| --- | --- | --- |
| PGPRB-8194 | GTGCTGCAAGGCGATTAAGT | Sequencing of inserts in pAKE604 |
| PGPRB-8195 | GAGCGCAACGCAATTAATGT | Sequencing of inserts in pAKE604 |
| PGPRB-10188 | GCCTGACTTCTGGCCACGGTCTGAATTACGCCCA | Cloning of *pmnH* in pET28a |
| PGPRB-10189 | TGGGCGTAATTCAGACCGTGGCCAGAAGTCAGGC | Cloning of *pmnH* in pET28a |
| PGPRB-10192 | TGGCTACCATGGGAACTATCGTTCTTCCACCCATCGTC | Cloning of *pmnH* in pET28a |
| PGPRB-10211 | TGGCTACTCGAGGTTAAGAATCAGGTTGTTGATCTCATCGA | Cloning of *pmnH* in pET28a |
| PGPRB-10249 | TGGCAGCAGCCAACTCAGCTT | Sequencing of inserts in pET28a |
| PGPRB-10250 | TATAGGCGCCAGCAACCGCA | Sequencing of inserts in pET28a |
| PGPRB-10255 | GCTCACTCATTAGGCACCC | Sequencing of inserts in pJB3Tc20 |
| PGPRB-10256 | GGTAACGCCAGGGTTTTC | Sequencing of inserts in pJB3Tc20 |
| PGPRB-10273 | AACAAGCCAGGGATGTAACG | Flanking sequences after plasposon rescue (pRL27) |
| PGPRB-10274 | CAGCAACACCTTCTTCACGA | Flanking sequences after plasposon rescue (pRL27) |
| PGPRB-10338 | TGGCTAAAGCTTCCAACAAGTGCTGATGCGCC | Construction of a *fiuA* knockout in F113 |
| PGPRB-10339 | TGGCTAGGATCCCATACGGCGGCTCCTGAATG | Construction of a *fiuA* knockout in F113 |
| PGPRB-10340 | TGGCTAGGATCCTAAACGGTTAGCATTTGCACAAATCTTTTGAGG | Construction of a *fiuA* knockout in F113 |
| PGPRB-10341 | TGGCTAGAATTCAAGGTGTGGGATGAATTGGGTTCG | Construction of a *fiuA* knockout in F113 |
| PGPRB-10367 | TGGCTAAAGCTTTTGAGAAATATTTCCATTCAGGAGCCGCC | Complementation of *fiuA* knockout in F113, cloning in pJB3Tc20 |
| PGPRB-10368 | TGGCTAGGATCCCCTCAAAAGATTTGTGCAAATGCTAACCGTTTAC | Complementation of *fiuA* knockout in F113, cloning in pJB3Tc20 |
| PGPRB-10369 | CGTGTTGAAATCCGCTGTG | Deletion validation of *fiuA* in F113 |
| PGPRB-10370 | ATTGGCCGAGGGCATTTC | Deletion validation of *fiuA* in F113 |
| LK-0011 | GTATCCGCCGCCAACGCCACCTACGATTCGATG | Construction of H224A in PmnH |
| LK-0012 | CATCGAATCGTAGGTGGCGTTGGCGGCGGATAC | Construction of H224A in PmnH |
| LK-0013 | GCCAACCACACCTACGCTTCGATGCCTGCCGGT | Construction of D227A in PmnH |
| LK-0014 | ACCGGCAGGCATCGAAGCGTAGGTGTGGTTGGC | Construction of D227A in PmnH |
| LK-0015 | TGGCTACTCGAGGAGCTCTTTGGTAACGGTAACTGC | Construction of ColN-domain deletion in PmnH |
| LK-0016 | TGGCTACTGCAGCGTACAACAGGATTTCCGAGCAATG | Cloning of *imnH* in pJB3Tc20 |
| LK-0017 | TGGCTAGAATTCTGGCGTGAAGCTAGTGCTGT | Cloning of *imnH* in pJB3Tc20 |
| LK-0047 | TGGCTAAAGCTTTTGAGAAATATTTCCATTCAGGAGCC | Complementation of *fiuA* transposon mutants in LMG 1794, cloning in pJB3Tc20 |
| LK-0048 | TGGCTAGGATCCCATTTTCTTGCCTTCAGACACACC | Complementation of *fiuA* transposon mutants in LMG 1794, cloning in pJB3Tc20 |

^a^ Restriction sites incorporated in the primers are underlined: GGATCC, BamHI; GAATTC, EcoRI; AAGCTT, HindIII; CCATGG, NcoI; CTGCAG, PstI; CTCGAG, XhoI.
